# Michigan State Veterinary Medical Association

**Published:** 1898-03

**Authors:** William Jopling

**Affiliations:** Secretary


### MICHIGAN STATE VETERINARY MEDICAL ASSOCIATION.

THE sixteenth annual meeting was held in the parlors of the Hotel Wentworth, Lansing, Mich., February 8 and 9, 1898. First Vice-President Dr. J. Black, of Richmond, called the meeting to order at 2.30 P.M.

The following members answered roll-call: Drs. J. Hawkins, D. Cummings, E. A. A. Grange, W. J. Byers, B. C. McBeth, William Jopling, W. W. Thorburn, G. W. Dunphy, James J. Joy, A. Campbell, Judson Black, F. M. Blatchford, H. F. Palmer, A. McKercher, W. S. Hamilton, L. A. Grinnell, D. M. Waldo, Edward T. Handy. Minutes of the last annual meeting were read and approved.

Acting President Dr. J. Black delivered quite a lengthy address to the Association. He said that reports from many of our colleagues in this State and other places do not reassure us that practice, on the whole, has improved much over last year, but he thought that the conditions were favorable for improvement in the near future. He put in a strong plea for the Association, urging the members to redouble their efforts in its behalf. He suggested that the Committees on Intelligence and Education and on Diseases endeavor to make full reports at our meetings. These reports form an important part of our programme, and if some extra effort was made by those committees in gathering material from the time of one meeting till the date of the next, very interesting papers would be the result.

Dr. Black said further that we have to our credit, the past year, one act that has amply repaid all trouble and expense, viz, the hanging on the fence the hide of one "Dr." Coester, erstwhile State Veterinarian. He had been appointed by Governor Pingree after this Association, at its last annual meeting, unanimously requested him to reappoint Prof. Grange. The Association being ignored entirely in the matter, were naturally filled with righteous indignation, and proceeded to make a "kick;" but the Governor would not reconsider the matter, and the Senate was appealed to, but in vain. It was only after said Coester, by indisputable evidence, was proved to be a "quack," a graduate of no known college, and therefore not eligible to hold the office, that our turn came, and we were successful in having one of our members appointed to the position.

The subject of milk- and meat-inspection has as yet received little or no consideration from the health authorities of this State compared with such action in other States. He thought it was our duty as veterinarians to sound the alarm and impress upon the health authorities the necessity of such legislation.

Communications were read from Dr. W. McQueen, of Oxford; Dr. H. M. Gahn, St. Johns; Dr. J. W. Ferguson, Bay City; Dr. D. P. Yonkerman, Kalamazoo; Dr. George Waddle, Hastings; Dr. J. C. Whitney, Hillsdale; Dr. S. Brenton, Detroit, expressing regret at their inability to be present. Also a communication from Dr. W. H. Hoskins, of Philadelphia, in which he expressed thanks for programmes of our meeting, and asked for a report of the proceedings. Also an invitation from Mayor William C. Mayberry, of Detroit, and the Detroit Convention and Business Men's League, to hold our next annual meeting in Detroit.

The several communications read were received and placed on file.

The report of the Secretary was read and accepted.

The report of the Treasurer was read and referred to the Finance Committee, as were also the bills presented.

Prof. Grange reported for the Committee on Intelligence and Education. He brought out the following points: That nearly all veterinary colleges had adopted the three-year-course plan. That tetanus-antitoxin was a prophylactic, but not a curative agent. That glanders had nearly disappeared in this State. He read a communication that was a translation from German, speaking of the prevalence of tetanus in certain places there, the conclusion being that it was unsafe to perform any operations without first treating the patient with antitoxin. This paper was of such interest that a motion was made and passed that Prof. Grange have the same sent to the veterinary journals for publication. Prof. Grange also suggested the idea of a circulating library for the members, and gave plans as to how same might be operated; the various veterinary journals might also be read by a larger number, if some co-operative plan could be arranged, as the pocket-book of only a few could afford to subscribe for all the journals. He also thought it would be well for our Association to have a copy of the *Veterinary Blue-Book,* edited by Dr. Huidekoper. A good discussion followed, participated in by Drs. Hawkins, Hamilton, Palmer, Dunphy, Campbell, and others.

Dr. J. Hawkins reported for the Committee on Diseases, tetanus and parturient apoplexy being discussed freely.

Owing to an injury, Dr. Giffin, chairman of the Committee on Legislation, was not present, but presented his report through Dr. Hawkins. Upon motion the report was accepted and the committee discharged.

Adjournment was taken till 7.30 P.M., at which time the meeting was called to order by Dr. Black.

The Finance Committee made their report, which showed that everything was correct with the Treasurer's books. The report was accepted.

Dr. George W. Dunphy, State Veterinarian, read a paper on "Azoturia," which was freely discussed.

Dr. J. Hawkins gave his experience with hog-cholera. He said it was assuming alarming proportions in the State. The discussion following was interesting. The disease is apparently incurable, and thorough disinfection during an outbreak and following the same was recommended.

Prof. C. D. Smith, of the Agricultural College, was introduced at this time by Prof. Grange. He made a pleasant address, and invited the Association to visit the college and experiment station in the morning. The invitation was accepted and arrangements made to take the street-car at 8 A.M.

The question of legislation was taken up. Dr. Hawkins read the bill as presented to the last Legislature. A slight amendment was made to it, and the old committee, consisting of Drs. Giffin, Black, and Ferguson, with the addition of Dr. G. W. Dunphy, were appointed to take the same in hand and have it presented to the Legislature at next session as early as possible.

The question of members being reinstated who were suspended for non-payment of dues was discussed, and a resolution passed that any suspended member applying for reinstatement can be so reinstated by paying all back dues up to the date of application for reinstatement.

The Association accepted an invitation from the Ohio Association to meet with them in joint session in Toledo, Ohio, next July. It was decided that, in case arrangements were made to meet the Ohio Association in July, arrangements be made to spend one day in Detroit before going to Toledo.

The question of asking the U. S. V. M. A. to hold a meeting in Detroit in the near future was also discussed.

Election of officers resulted as follows: President, Dr. J. Black, Richmond; First Vice-President, Dr. H. F. Palmer, Brooklyn; Second Vice-President, Dr. James J. Joy, Detroit; Third Vice-President, Dr. E. T. Handy, Eaton Rapids; Secretary and Treasurer, Dr. William Jopling, Owosso; Board of Directors, Drs. E. A. A. Grange, F. M. Blatchford, A. McKercher, James Drury, D. M. Waldo, and J. W. Ferguson. Standing Committees—Intelligence and Education: Drs. E. A. A. Grange, J. Hawkins, and D. Cummings. Diseases: Drs. G. W. Dunphy, W W. Thorborn, and J. J. Joy. Finance: Drs. A. Campbell, L. A. Grennell, and W. S. Hamilton. Legislation: Drs. W. A. Giffin, J. Black, J. W. Ferguson, and G. W. Dunphy.

Essays and papers for next meeting: Dr. H. F. Palmer, "Milk- and Meat-inspection;" Dr. E. T. Handy, "Parturient Apoplexy;" Prof. E. A. A. Grange, "Disinfection;" Dr. W. W. Thorburn, "Electricity as Applied to Animals;" Dr. S. Brenton, "Wry Tail;" Dr. F. M. Blatchford, "Selected."

Adjournment to meet at 8 o'clock A.M., Wednesday, February 9th, at which time all assembled and went to the Agricultural College, where two hours were very pleasantly spent in looking over the various departments.

From 10.30 to 12.30 a clinic was held at the infirmary of Dr. Thorburn. Drs. Joy, of Detroit; Dunphy, of Quincy; and Campbell, of Jackson, being the principal demonstrators.

After dinner a short session was held before taking final adjournment.

A vote of thanks was given Prof. Smith and the fraternity of the Agricultural college for the very courteous and considerate attention accorded us while at the college.

A vote of thanks was tendered Mr. and Mrs. Wentworth, our host and hostess, for their kindness and attention, which aided materially in making our stay in the capital very pleasant.

WILLIAM JOPLING,
Secretary.

